# Physical activity and beta-amyloid pathology in Alzheimer's disease: A sound mind in a sound body

**DOI:** 10.17179/excli2017-475

**Published:** 2017-06-28

**Authors:** Khadije Ebrahimi, Alireza Majdi, Behrouz Baghaiee, Seyed Hojjat Hosseini, Saeed Sadigh-Eteghad

**Affiliations:** 1 Department of Sports Science and Physical Education, Marand Branch, Islamic Azad University, Marand, Iran; 2 Neurosciences Research Center, Tabriz University of Medical Sciences, Tabriz, Iran; 3 Department of Sports Science and Physical Education, Jolfa Branch, Islamic Azad University, Jolfa, Iran; 4 Department of Physiology and Pharmacology, School of Medicine, Zanjan University of Medical Sciences, Zanjan, Iran

**Keywords:** physical activity, Alzheimer's disease, beta-amyloid, prevention

## Abstract

Alzheimer's disease (AD) is the most common type of dementia worldwide. Since curative treatment has not been established for AD yet and due to heavy financial and psychological costs of patients' care, special attention has been paid to preventive interventions such as physical activity. Evidence shows that physical activity has protective effects on cognitive function and memory in AD patients. Several pathologic factors are involved in AD-associated cognitive impairment some of which are preventable by physical activity. Also, various experimental and clinical studies are in progress to prove exercise role in the beta-amyloid (Aβ) pathology as a most prevailing hypothesis explaining AD pathogenesis. This study aims to review the role of physical activity in Aβ-related pathophysiology in AD.

## Introduction

Alzheimer's disease (AD) is the most prevalent form of dementia and is an age-related neurodegenerative disorder leading to loss of memory and learning dysfunction in mid to late life (Sadigh-Eteghad et al., 2014[74]). AD is characterised by an inexorable loss of neurons, particularly in the hippocampus and cerebral cortex resulting in cognitive dysfunction. From the histopathological point of view, AD progression is most commonly associated with extracellular deposition of beta-amyloid (Aβ) peptides in the brain which forms senile plaques (SPs) and tau protein which creates neurofibrillary tangles (NFT) (Sadigh-Eteghad et al., 2014[75]; Serrano-Pozo et al., 2011[79]). 

Most experts agree that AD develops as a result of numerous factors rather than a single cause which exert their effects nearly twenty years before AD symptoms appear. The “amyloid cascade” hypothesis is the most common and accepted theory and proposes that assembly of Aβ and toxic effects of its oligomeric forms cause AD, while other pathological changes are downstream to the continuing aggregation of Aβ (Correia et al., 2011[11]). Also, alteration in Aβ regulatory factors such as amyloid precursor protein (APP), beta-site APP-cleaving enzyme 1 (BACE1), presenilin (PS) 1 or 2, apolipoprotein E (APOE), neprilysin (NEP) and insulin-degrading enzyme (IDE), could play an important role in AD initiation and progression (Dong et al., 2012[17]). 

Prevention in the earliest stages of AD is probably the most effective way to reduce the prevalence of this disorder (Riedel, 2014[71]). This could define interventional trials in AD and draw particular attention to AD-preventing activities (Vellas et al., 2011[90]). Moreover, international efforts have aimed to develop preventive strategies due to the lack of disease-modifying therapies (Ahlskog et al., 2011[2]).

There are some preventable lifestyle-related risk factors such as sedentary lifestyle and lack of physical activity which increase the risk of dementia and AD (Sink et al., 2015[80]; Prakash et al., 2014[66]). A rapidly growing body of evidence strongly suggests that physical exercise may attenuate cognitive impairment and reduce the risk of AD via a variety of mechanisms (Ahlskog et al., 2011[2]). However, these findings are not universal and some studies could not prove this association (Sink et al., 2015[80]) (Table 1(Tab. 1); References in Table 1: Krell-Roesch et al., 2016[42]; Reiter et al., 2015[70]; Sink et al., 2015[80]; Train the Brain Consortium, 2017[88]; Tapia-Rojas et al., 2016[87]; Prakash et al., 2014[66]; Deeny et al., 2012[14]; Raichlen and Alexander, 2014[68]; Liu et al., 2013[49]; Um et al., 2008[89]; Cho et al., 2010[8]; Nichol et al., 2008[62]; Herring et al., 2011[32]; Xiong et al., 2015[92]; McConlogue et al., 2007[58]; Cho et al., 2015[7]; Kang et al., 2013[38]; Zhao et al., 2015[98]; Diegues et al., 2014[16]; Nichol et al., 2008[62]; Zhao et al., 2007[99]; Kim et al., 2011[41]). Evidence suggests that physical activity may attenuate cognitive impairment through Aβ-independent mechanisms such as a decrease in the activated microglia and increase in the brain-derived neurotrophic factor (BDNF) positive cells (Xiong et al., 2015[92]). However, Aβ is one of the most important role players in AD pathology and prevention (Sadigh-Eteghad et al., 2014[75]). It seems that exercise improves learning and memory, increases hippocampal neurogenesis, plasticity as well as volume, and decreases Aβ load and plaque deposition in the central nervous system (CNS) (Hötting and Röder, 2013[34]). Therefore, this study aims to gather and review the old and most recent data on the role of physical activity in Aβ-related pathophysiology of AD. 

## Beta-Amyloid

One of the theories explaining the pathology of AD is "amyloid cascade hypothesis" that was first purposed in 1992 (Hardy and Higgins, 1992[30]). According to this popular theory, the primary pathological event in AD includes Aβ peptide production and deposition in the brain parenchyma, which leads to the formation of SPs, NFTs, death of neurons, and eventually dementia (Armstrong, 2011[3]; Karran et al., 2011[39]). 

Aβ peptide can be detected in human cerebrospinal fluid (CSF) in various forms including Aβ_40_ and Aβ_42_. The latter is the most common type and is more toxic than the former (Giedraitis et al., 2007[27]; Yan and Wang, 2006[95]). Hence, in most familial AD cases , Aβ_42_/Aβ_40_ ratio is elevated in the brain which predisposes individuals to AD (Karran et al., 2011[39]; Kumar‐Singh et al., 2006[43]). Alternatively, the active and likely the most toxic Aβ species are the soluble oligomers which form fibrils as the main component of Aβ plaques (Jagust and Mormino, 2011[35]).

It has been found that exercise exerts its anti-Aβ effects through various mechanisms. Evidence suggests that exercise decreases soluble Aβ_42_ protein in AD mice brain (Zhao et al., 2015[98]). Physical activity also significantly decreases soluble Aβ_40_ and fibrillar Aβ in the aged transgenic AD mice (Nichol et al., 2008[62]). Similarly, a randomised controlled trial showed that high-intensity aerobic exercise in patients with MCI decreases plasma concentrations of Aβ_42 _(Baker et al., 2010[5]). Further, studies using transgenic AD mice have shown that exercise causes a reduction in Aβ load and APP metabolism in brain (Cho et al., 2010[8]; Leem et al., 2009[46]; Um et al., 2008[89]).

Physical activity also results in a reduction in extracellular Aβ which appears to be mediated by a change in the processing of APP (Adlard et al., 2005[1]). Besides, preventive physical stimulation before disease onset alters APP processing and increases Aβ degradation (Herring et al., 2011[32]). Additionally, Aβ-dependent neuronal cell death in the hippocampus of transgenic AD mice markedly decreases following exercise suggesting the inhibitory potential of exercise in both Aβ_42_ and neuronal death pathways. Furthermore, exercise is known to enhance hippocampus-associated memory and amygdala-associated neuronal function and serve as a mean to delay the onset of AD in transgenic mice (Lin et al., 2015[48]). It was recently reported that voluntary wheel running decreases astrogliosis and increases neurogenesis in the hippocampus of runner AD mice (Tapia‐Rojas et al., 2016[87]). 

These results suggest that exercise represents a potential therapeutic strategy for AD by reducing beta-amyloidogenesis and brain beta-amyloid burden (Um et al., 2008[89]). 

## Beta-Site APP Cleaving Enzyme 1

APP is an integral membrane glycoprotein that is expressed in the CNS. APP is sequentially cleaved by β-secretase (BACE1) and γ-secretase to generate Aβ (Cole and Vassar, 2007[9]; Farzampour et al., 2016[25]). Increased Aβ production by sequential cleavage of APP by β and γ-secretases contributes to the etiological basis of AD (Joshi and Wang, 2015[36]). 

BACE1 is a protease and a member of pepsin family with higher concentration in the neurons which cleaves APP at β-site and increases Aβ level in neurons (Yan and Vassar, 2014[94]). BACE1 concentration is roughly two-fold higher in the brain of AD patients compared with a healthy non-demented brain (Yan and Vassar, 2014[94]). Also, BACE1 inhibitors can decrease Aβ production in neurons (McConlogue et al., 2007[58]). 

It has been shown that BACE1 expression diminished in triple transgenic mice following a treadmill running protocol (Cho et al., 2015[7]). Also, there is evidence that, treadmill exercise may modulate APP processing through BACE1 suppression and decrease Aβ generation and deposition in neurons (Kang et al., 2013[38]; Liu et al., 2013[49]). Furthermore, it was shown that treadmill exercise significantly improves learning and memory performance in d-galactose-induced ageing in rats by repression of Aβ_42_ protein levels, through down-regulation of BACE1 mRNA in the rat hippocampus (Yu et al., 2013[96]). Finally, exercise can reduce BACE1 content which is accompanied by a reduction in Akt, ERK, and signalling in the cortex, indicating a decline in cellular stress (MacPherson et al., 2015[52]).

## Presenilin 1/2

AD can also result from autosomal dominant mutations in PS1 and PS2, both of which are homologous proteins. PS1 is part of the γ-secretase complex that processes APP, but PS2 attaches to the complex and helps to stabilise it (Kang et al., 2013[38]; Karran et al., 2011[39]; Zhang et al., 2013[97]). Autosomal dominant mutations in PS1 or PS2 change APP processing towards Aβ_42_ (Hardy and Selkoe, 2002[29]; Scheuner et al., 1996[76]). It is now broadly recognised that γ-secretase constitutes at least four different proteins: PS1, PS2, nicastrin, and Aph-1 (Edbauer et al., 2003[20]) where PS is the catalytic core of the complex (De Strooper, 2003[12]). Mutations in PS1/2 mainly account for early onset familial AD (Larner and Doran, 2006[44]). Many of these mutations enhance the relative aggregation of more neurotoxic Aβ_42_ peptides (Hardy and Selkoe, 2002[29]).

It has been proved that exercise inhibits PS2 mutation-induced memory impairment and activation of β-secretase, and reduces Aβ_42_ depositions in the cortex and hippocampus of aged PS2 mutant mice (Kang et al., 2013[38]). Consistently, physical activity significantly reduces the PS1 expression and APP phosphorylation in the hippocampus of APP/PS1 mice which is accompanied by a remarkable decrease in the aggregation of Aβ (Liu et al., 2013[49]).

## Apolipoprotein E

APOE is a class of apolipoprotein predominantly found in CNS and is normally synthesised and produced by astrocytes and microglia, but neuropathologically by neurons (Grehan et al., 2001[28]; Leoni et al., 2010[47]; Mahley, 2016[54]; Xu et al., 2000[93]). There are three common APOE alleles in human: APOE2, APOE3 and APOE4. While transporting cholesterol is a primary function, APOE also regulates Aβ metabolism (Kanekiyo et al., 2014[37]). APOE protein exists in senile plaques and can bind to the hydrophobic Aβ peptides (Strittmatter et al., 1993[86]) and affects the formation, agreeability, or clearance of extracellular Aβ (Honjo et al., 2012[33]). 

Alterations in lipid homoeostasis could serve as common denominator for APOE and Aβ dysfunctions in AD (Poirier, 2000[65]). Further, series of experiments demonstrate that cognate proteins which mediate the clearance of Aβ with APOE2, APOE3 and APOE4 become increasingly less effective at clearing Aβ (Karran et al., 2011[39]). Among these three human APOE isoforms, APOE4 increases the risk of AD. Heterozygous APOE-ε4 carriers (ε2/ε4 or ε3/ε4) have 3-4 times higher risk of developing AD (Honjo et al., 2012[33]; Jagust and Mormino, 2011[35]; Mahley et al., 2006[55]). It has been shown that APOE-ε4 allele highly involves in the Aβ deposition and is an important genetic cause for late-onset AD (Ba et al., 2016[4]; Schmechel et al., 1993[77]; Rebeck et al., 1993[69]). Alternatively, APOE4 can change Aβ_40_ into an Aβ_42_-like structure or more aggregated APOE4-Aβ_40_ complex (Kim et al., 2009[40]). 

Recently, studies have revealed a novel interaction between APOE status and exercise engagement (Head et al., 2012[31]; Low et al., 2010[50]; Prakash et al., 2014[66]; Smith et al., 2016[81]). It has been shown that regular physical activity may prevent or delay symptoms of dementia and AD, especially among persons with APOE ε4 allele (Rovio et al., 2005[72]). Schuit et al. found that the risk of cognitive decline was higher in participants who reported less than 1 hour of physical activity daily and were also APOE4 carriers (Schuit et al., 2001[78]). It is indicated that amyloid levels were higher in the brain of physically inactive APOEε4 allele carriers but were lower in physically active ε4 carriers that were tantamount to those of non-carriers (Head et al., 2012[31]). Similarly, other neuroimaging studies have reported that physical activity could be more effective in ε4 carriers than in non-carriers (Deeny et al., 2012[14]; Smith et al., 2011[82]). Also, there is an inverse association between dementia risk and physical activity in APOE4 non-carriers which is lower than APOE4 carriers (Luck et al., 2014[51]; Podewils et al., 2005[64]; Rovio et al., 2005[72]). 

Besides Aβ-dependent pathways, physical activity can improve cognitive function in adult ε4 carriers through other mechanisms such as improved perfusion and neurogenesis as well as neuroprotective processes (Raichlen and Alexander, 2014[68]). 

Smith et al. suggested that APOE genotype knowledge can play an essential role in providing recommendations on physical activity for elderly as a tool to prevent future cognitive decline and brain atrophy (Smith et al., 2014[83]). In general, these results propose that people who are genetically more susceptible to AD benefit profoundly more from effects of physical activity.

## Neprilysin/Insulin-Degrading Enzyme

Neprilysin (NEP) and insulin degrading enzyme (IDE) are part of degrading enzymes, which determine Aß concentration (Miners et al., 2008[59]). Due to age-related genetic mutation, the activity of these enzymes decreases which predisposes the patient to AD (Eckman and Eckman, 2005[18]; Marr et al., 2004[57]). Overall, growing evidence suggests that NEP and IDE levels decrease in AD leading to the disruption of the balance between production and clearance of Aβ in AD (Dong et al., 2012[17]).

NEP, known as a thermolysin-like zinc metallopeptidase, is a type-II integral membrane protein mostly found in kidney (El-Amouri et al., 2008[22]; Gayathiri et al., 2014[26]). This enzyme is located pre and postsynaptically in neuronal cells and is able to cleave monomeric and oligomeric forms of Aβ from brain (El-Amouri et al., 2008[22]; Miners et al., 2006[60]). Overexpression of NEP has been reported to protect hippocampal neurons from Aβ-mediated toxicity (El-Amouri et al., 2007[21]). 

IDE is also a zinc metalloendopeptidase that is highly expressed by microglial cells and neurons in the brain and cleaves extracellular Aβ (Miners et al., 2008[59]; Pivovarova et al., 2016[63]). In addition to its capability to degrade Aβ extracellularly in the brain, IDE is capable of removing cytoplasmic products of APP (Bernstein et al., 1999[6]; Edbauer et al., 2002[19]; Wang et al., 2010[91]). Some studies reported that IDE levels, as well as Aβ degradation, tend to decrease in the hippocampus and CSF of patients with AD which is more significant in APOE e4 carriers (Cook et al., 2003[10]; Del Campo et al., 2015[15]; Son et al., 2016[85]). Membrane-bound IDE levels and its activity significantly decrease in MCI patients and seem to have further decrease during the transition from MCI to AD (Zhao et al., 2007[99]). 

Moore et al. (2016[61]) showed that NEP and IDE activity and mRNA expression level increase in a dose-dependent manner in the cortex and hippocampus by low and high-intensity exercise training. The increase was higher for NEP, which is the most active Aβ degrading enzyme. Also, their data demonstrated that exercise reduces extracellular soluble Aβ in the brains of Tg2576 mice in a dose-dependent manner. Additionally, the swimming exercise markedly increased expression of IDE in the rat model of non-insulin-dependent diabetes mellitus (Kim et al., 2011[41]). Voluntary exercise in high-fat-diet-induced Aβ deposition and memory deficit in APP transgenic mice ameliorates Aβ deposition by strengthening the activity of NEP (Maesako et al., 2012[53]). Consistently, Lazarov et al. (2005[45]) observed that environmental enrichment with running wheel significantly elevates the activity of NEP in the brain. 

## Physical Activity and Cognitive Decline in AD/MCI

Some factors affect physical activity impact on the prevention of cognitive decline in AD and MCI. 

First, the timing and intensity of physical activity have an important effect on prevention of AD and mild cognitive impairment (MCI). It has been shown that even light physical activity in mid- and late life is linked to a lower risk of MCI. Several mechanisms may be responsible for this effect including increased production of neurotrophic factors and neurogenesis, increased cerebral blood flow, and reduced risk of cardiovascular diseases (Krell‐Roesch et al., 2016[42]). Also, it is proven that moderate-intensity regular exercise is associated with increased cortical thickness and enhanced cognitive function through improved cardiorespiratory fitness in MCI patients (Reiter et al., 2015[70]).

Second, the additive effects of cognitive training/enrichment on physical activity against AD and MCI have been shown in some studies. In a study by Sacco et al., it was shown that addition of cognitive tasks such as single reaction time and an inhibition task (Go-no-Go) enhances physical activity positive effects on cognitive performance in AD and MCI patients (Sacco et al., 2016[73]). This might result from modifying effects of training tasks on blood oxygen level dependent (BOLD) response within task-related brain regions (Train the Brain Consortium, 2017[88]). 

These factors can be used to intensify improving effects of physical activity on cognitive decline in dementia patients. 

## Physical Activity and Aβ-Independent Mechanisms

Several studies have shown that physical activity can also improve cognitive function in AD and MCI patients through Aβ-independent mechanisms. It has been shown that physical activity increases neurotrophic factors such as BDNF levels in the brain and through their neuroprotective effects improve cognition in AD (Erickson et al., 2012[24]). Also, regular physical activity acts as a preconditioner against oxidative stress and reactive oxygen species production (ROS). Physical activity reduces ROS-induced protein damage and controls the activity of redox-sensitive transcription factors (Radak et al., 2007[67]) which are all involved in neurodegeneration and dementia (Majdi et al., 2016[56]). Physical activity also decreases neuroinflammation in the brain. In a study by Kang et al., it was demonstrated that treadmill exercise reduces neuronal cell death and TNF-α as well as IL-1α expression levels in aged PS2 mutant mice and thus decreases Aβ-induced inflammatory response (Kang et al., 2013[38]) that is involved in dementia (Majdi et al., 2016[56]). Further, it has been found that endothelial function is impaired in patients with AD and together with lipid and lipoprotein metabolism may be part of vascular contributions to cognitive impairment and dementia (VCID) (Dede et al., 2007[13]; Snyder et al., 2015[84]). Physical activity enhances endothelial nitric oxide synthase (eNOS) activity in the brain vasculature, increases blood flow and decreases subsequent cerebral damage (Endres et al., 2003[23]). 

## Conclusion

AD progression is mostly associated with extracellular deposition of Aβ peptides in brain. Also, alteration in Aβ regulatory factors such as BACE1, PS1/2, NEP and IDE enzymes, and APOE-ε4 allele could play an essential role in AD progression. In addition, lifestyle-related risk factors such as sedentary life pattern and lack of physical activity increase the risk of dementia and AD. 

**Figure 1:**
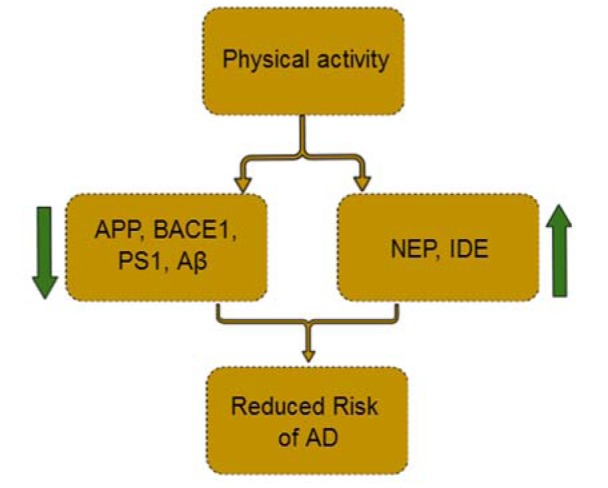
Mechanisms of physical activity in prevention of Alzheimer's disease risks

The most effective way to protect brain and reduce risk of AD is likely prevention in preclinical stages. It seems that physical activity especially aerobic exercise is one of these preventing factors. Evidence suggests that physical activity can improve cognitive function by various Aβ-dependent and independent mechanisms including reducing Aβ_42_, APP, BACE1 and PS1 level and on the other hand, elevating NEP and IDE activities (Figure 1(Fig. 1)). Consistently, physical activity may reduce the risk of AD, especially among APOE ε4 allele carriers. Overall, physical activity could have beneficial effects on prevention or delay of symptoms of AD. Nevertheless, the optimum activity condition with the best efficacy and exact mechanisms behind should be clarified in future studies.

## Figures and Tables

**Table 1:**
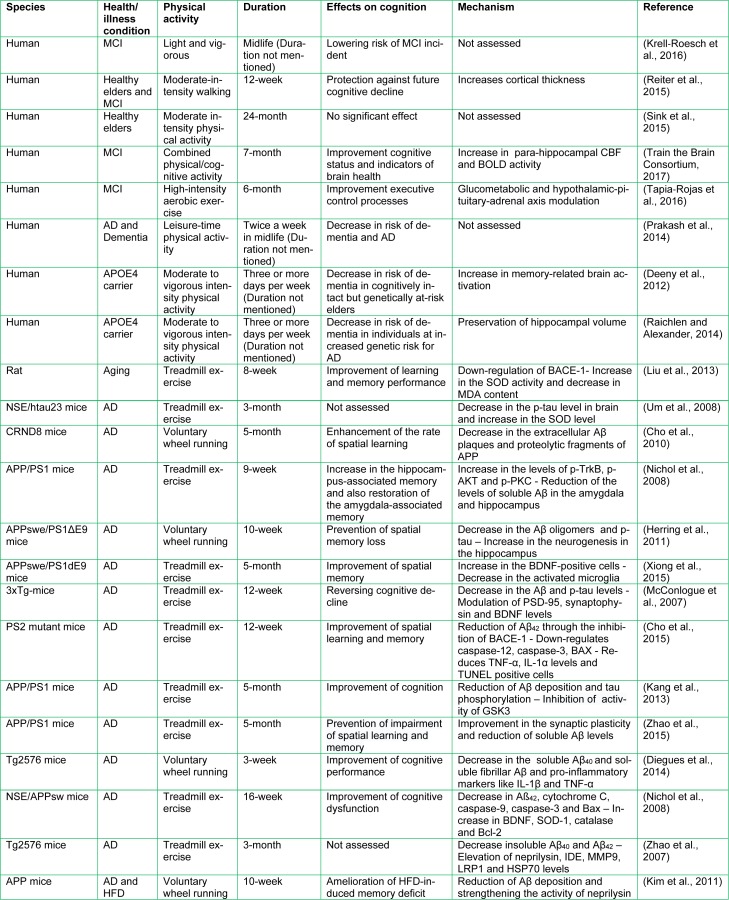
Effects of physical activity on cognition and related mechanisms in clinical and experimental studies. AD, Alzheimer's disease; MCI, mild cognitive impairment; CBF, cerebral blood flow; BOLD, blood-oxygen-level-dependent; BACE-1, β-site amyloid precursor protein cleaving enzyme 1; PSD95, postsynaptic density protein 95; SOD, superoxide dismutase; MDA, malondialdehyde; Aβ, beta-amyloid; BDNF, brain-derived neurotrophic factor; IL, interleukin; TNF-α, tumour necrosis factor-α; MMP9, matrix metallopeptidase 9; LRP1, lipoprotein receptor-related protein 1; HSP70, heat shock protein 70; IDE, insulin degrading enzyme; HFD, high-fat diet.
